# Is intraindividual reaction time variability an independent cognitive predictor of mortality in old age? Findings from the Sydney Memory and Ageing Study

**DOI:** 10.1371/journal.pone.0181719

**Published:** 2017-08-09

**Authors:** Nicole A. Kochan, David Bunce, Sarah Pont, John D. Crawford, Henry Brodaty, Perminder S. Sachdev

**Affiliations:** 1 Centre for Healthy Brain Ageing (CHeBA), School of Psychiatry, University of New South Wales (UNSW) Australia, Sydney, NSW, Australia; 2 Neuropsychiatric Institute, Prince of Wales Hospital, Randwick, NSW, Australia; 3 School of Psychology, Faculty of Medicine and Health, University of Leeds, Leeds, United Kingdom; 4 Dementia Collaborative Research Centre–Assessment and Better Care (DCRC-ABC), School of Psychiatry, UNSW Australia, Sydney, NSW, Australia; Nathan S Kline Institute, UNITED STATES

## Abstract

Intraindividual variability of reaction time (IIV_RT_), a proposed cognitive marker of neurobiological disturbance, increases in old age, and has been associated with dementia and mortality. The extent to which IIV_RT_ is an independent predictor of mortality, however, is unclear. This study investigated the association of IIV_RT_ and all-cause mortality while accounting for cognitive level, incident dementia and biomedical risk factors in 861 participants aged 70–90 from the Sydney Memory and Ageing Study. Participants completed two computerised reaction time (RT) tasks (76 trials in total) at baseline, and comprehensive medical and neuropsychological assessments every 2 years. Composite RT measures were derived from the two tasks—the mean RT and the IIV_RT_ measure computed from the intraindividual standard deviation of the RTs (with age and time-on-task effects partialled out). Consensus dementia diagnoses were made by an expert panel of clinicians using clinical criteria, and mortality data were obtained from a state registry. Cox proportional hazards models estimated the association of IIV_RT_ and mean RT with survival time over 8 years during which 191 (22.2%) participants died. Greater IIV_RT_ but not mean RT significantly predicted survival time after adjusting for age, sex, global cognition score, cardiovascular risk index and apolipoprotein ɛ4 status. After excluding incident dementia cases, the association of IIV_RT_ with mortality changed very little. Our findings suggest that greater IIV_RT_ uniquely predicts shorter time to death and that lower global cognition and prodromal dementia in older individuals do not explain this relationship.

## Introduction

In addition to average performance level, there is an increasing focus in ageing research on intraindividual variability or inconsistency in cognitive performance. Such variability in performance is often measured by the trial-to-trial within-person variation in reaction times (RT) on a single cognitive task and is known as intraindividual reaction time variability (IIV_RT_). IIV_RT_ has received considerable attention as a useful indicator of neurobiological disturbance [1]. Consistent with this, several studies indicate that IIV_RT_ is greater in older age [2] and in a variety of neuropathological conditions of old age including mild cognitive impairment [3], dementia [4] and Parkinson’s disease [5]. Additionally, associations have been found with measures of brain integrity, including white matter hyperintensities [6], brain connectivity [7], and dopaminergic neuromodulation [8].

Our present interest is whether this measure can predict mortality in old age. It is possible that neurobiogical changes that are related to eventual mortality are captured by variability measures and are present many years in advance. A few studies have reported that increased variability predicts mortality up to 19 years before eventual death in older populations [9–11] but it is unknown whether this association is independent of general age-related cognitive decline, an established risk factor for mortality [12, 13]. Moreover, the potential influence of incipient dementia on this relationship [14] has not been addressed adequately in previous studies that have used unreliable means of case identification (e.g., ‘questionable dementia’ written on death certificates [9], dementia screening measures with low sensitivity [10], or have not attempted to remove dementia cases [11]). There is preliminary support for the relative importance of IIV_RT_ as a predictor of mortality over the more basic measure of mean RT from the same cognitive task [9, 10] and this is worthy of further investigation particularly in the context of the potential influence of global cognitive level and prodromal dementia on these relationships.

Hence, IIV_RT_ warrants investigation as a specific predictor of impending death in older age independently of global cognitive level, other mortality risk factors and speed (mean RT from the same task) and prodromal dementia using robust clinical diagnoses in the years before death. Therefore, the aim of this study was to investigate the association of IIV_RT_ with mortality over 8 years in a large, well-characterised population-based cohort of older adults aged 70 years and over, taking into account general cognitive function as assessed by a battery of psychometric tests, other mortality risk factors including demographics, cardiovascular risk and apolioprotein ɛ4 status, and dementia diagnosis based on DSM-IV [15].

## Materials and methods

### Participants

Participants were drawn from the Sydney Memory and Ageing Study (MAS), a longitudinal study of community dwelling older adults recruited through the Australian electoral roll, aged 70 to 90 years at baseline [16]. Wave 1 MAS participants were recruited from September 2005 to November 2007. Exclusionary criteria were as follows: Mini-Mental State Score (MMSE) ≤ 24 [17] adjusted for age and education [18], or baseline diagnosis of dementia, schizophrenia, bipolar disorder, multiple sclerosis, motor neuron disease, developmental disability, and progressive malignancy. Of the remaining 1037 participants at baseline, we excluded 164 participants of non-English speaking background because validity of cognitive test performance for this group is questionable [19, 20], and 12 participants without RT data, leaving 861 for the the study sample. The study was approved by the Human Research Ethics Committees of the University of New South Wales and South East Sydney and Illawarra Area Health Service. Participants gave written informed consent.

### Reaction time measures

Simple and complex RT tasks were administered at baseline on a touch screen computer with millisecond accuracy [21]. For the simple RT task, participants had to touch a yellow square as quickly as possible, presented at 1, 2 or 4 second interstimulus intervals over a total of 36 trials over 2 blocks. For the complex RT, two coloured squares appeared vertically (red-red, yellow-yellow, red-yellow, yellow-red) at 3-second interstimulus intervals over a total of 40 trials over 2 blocks. Participants had to touch the upper square if the squares were the same colour or the lower square if the squares were different in colour. Practice trials prior to testing ensured that participants achieved four consecutive correct trials before they were allowed to continue. Processing of RT data and computation of metrics followed established procedures [21, 22]. For the IIV_RT_ measure, intraindividual standard deviation (*SD*) of RTs were computed using a regression procedure that partialled out effects of time-on-task (trial order) and age (and their interaction) from the individual RTs. The residuals obtained were then standardised and converted into T-scores, and finally an estimate of each individual’s standard deviation across the trials was computed. Mean RT was also computed. To obtain the most reliable estimates, linear-weighted composite scores for mean RT and IIV_RT_ were computed from the two RT tasks (Simple and Complex).

### Clinical and cognitive measures

Comprehensive assessments consisting of medical history, medical examination, neuropsychological measures and informant interviews were conducted by trained research psychologists at two-yearly intervals. Participants completed a battery of 10 psychometric tests measuring five major cognitive domains: attention/processing speed (Digit Symbol-Coding[23], Trail Making Test A[24]), memory (Rey Auditory Verbal Learning Test[24], Logical Memory[25], Benton Visual Retention Test[26]), language (Boston Naming Test[27], Category Fluency Test[24]), visuospatial (Block Design[28]) and executive abilities (Trail Making Test B[24], Letter Fluency Test[24]), details of which have been previously published [16, 19]. Participants received the neuropsychological battery at each wave they were present (ranging from 1–4 occasions) unless they were deemed too cognitively impaired (from wave 2 onward) in which case they were administered the Addenbrooke’s Cognitive Examination-Revised [29].

Wave 1 neuropsychological test data were used in the analyses. Raw scores were transformed to *z*-scores using baseline means and *SDs* of a subgroup of cognitively normal individuals. A global cognition score was calculated by averaging z-scores and transforming this so that the normal subgroup mean equals 0 and *SD* equals 1 (a higher score represents better performance). This cognitive composite score was used as a covariate in analyses to control for the known association of cognitive level with mortality [12]. The MMSE [17] and the National Adult Reading Test-Revised [30] were administered to measure current cognitive level and premorbid cognitive level respectively.

Other covariates previously linked to mortality included apolipoprotein ɛ4 (ApoE ɛ4) status [31] and cardiovascular disease risk [32], as well as age and sex. ApoE genotyping was obtained from genomic DNA extracted from peripheral blood or saliva [33] and ɛ4 carriers were compared to non-carriers (one or two ɛ4 alleles versus none). A cardiovascular disease risk score based on the Framingham Study [34] was derived from age, current smoking status, diabetic status, systolic blood pressure, total cholesterol level, high density lipoprotein level, and current hypertensive medication.

### Dementia and vital status ascertainment

At each study wave, cases were reviewed at case conference to reach a consensus on dementia diagnosis by a minimum of three clinicians from an expert multidisciplinary panel comprising of old age psychiatrists, neuropsychiatrists and clinical neuropsychologists. Dementia was diagnosed using DSM-IVcriteria [15] using all available clinical information, neuropsychological scores, and MRI when available (approximately half the sample) [16]. Vital status of participants, and cause and date of death were obtained from the New South Wales Registry of Births, Deaths and Marriages for 8 years after the study commenced.

### Statistical analyses

Analyses were performed using IBM SPSS Statistics 22.0. Baseline characteristics for deceased and surviving participants were compared using Chi-square for categorical variables, and Student’s t-test for continuous variables with the exception of the RT measures which were assessed using non-parametric tests (Mann-Whitney) as these variables were skewed. Bivariate correlations were assessed using Pearson’s correlations or Spearman’s for correlations between RT measures and other variables. Cox proportional-hazards models were used to examine the effects of baseline mean RT and IIV_RT_ on hazard rates of all-cause mortality. Time to event was computed as the number of years and months between each individual’s baseline assessment and a) date of death for decedents or b) the last assessment date for which surviving participants were known to be alive up to the end of the study period (July 2014). All survivors were included in the Cox analyses as right-censored. Mean RT and IIV_RT_ measures were transformed to *z* scores. Each RT measure was entered into separate models. The models were estimated without covariates (Model 1), then with covariates (e.g., age, sex, global cognition score, cardiovascular disease index, ApoE ɛ4 status) (Model2) and finally, a backward regression procedure was used with both RT measures included and all covariates to determine the best predictors of mortality (Model 3). All analyses were repeated after excluding incident dementia cases diagnosed over the 8-year period along with additional cases where dementia was listed as a cause of death on the Death Certificate.

## Results

Sample baseline characteristics based on vital status are shown in Table 1. Of 861 participants, 191 died (22.2%) within 8 years of follow-up with a mean survival time of 4.3 years (*SD* = 2.2). Decedants had significantly slower mean RT and greater IIV_RT_, lower general cognitive ability and a higher CVD risk score, and were older and more likely to be male than those who survived. Table 2 displays bivariate correlations between the main variables.

**Table 1:** Baseline characteristics based on vital status at 8 years.

Variable	n	Living (N = 670)	Deceased (N = 191)	Test Statistic	*p*-value
Age	861	77.87 (4.50)	81.38 (4.66)	*t*(859) = -9.45	**< .001**
Sex (% male)	861	41.2	54.5	*χ*^*2*^(1) = 10.59	**.001**
Education (years)	861	11.54 (3.37)	11.98 (3.96)	*t*(273.3) = -1.40	.16
Mean Reaction Time (ms)	861	743.19 (184.16)	778.69 (238.77)	*U*(859) = 52465.5	**< .001**
IIV_RT_	861	5.85 (3.76)	6.70 (3.51)	*U*(859) = 54698.0	**.001**
MMSE score^a^	861	28.57 (1.34)	28.54 (1.28)	*t*(859) = .32	.75
NART IQ^b^	847	107.61 (10.13)	107.34 (9.77)	*t*(845) = .31	.76
Global Cognition z-score^c^	860	-.43 (1.20)	-.98 (1.49)	*t*(262.4) = 4.65	**< .001**
CVD risk score	830	16.97 (3.37)	17.82 (3.47)	*t*(828) = -.3.00	**.003**
ApoE ɛ4 allele carrier^d^ %	816	23.5	21.3	*χ*^*2*^(1) = .367	.55

RT = reaction time; IIV_RT_ = intra-individual variability of reaction time.

^a^ MMSE = Mini-Mental State Examination Score, adjusted for age and education

^b^ NART IQ = National Adult Reading Test-Revised estimated IQ score

^c^ Composite score derived from baseline performance on a battery of 10 neuropsychological measures using the average of the z-scores for each test and transforming this so that the normal reference group has a mean equal to 0 and *SD* equal to 1 (a higher score represents better performance).

^d^compared to non-carrier

Median (interquartile range) is shown for Mean RT and IIV_RT_, mean (SD) for other variables.

*p*-values in bold indicate significance at 0.05 level.

**Table 2:** Correlations between age, cognitive measures CVD risk score.

	Mean RT	IIV_RT_	Cognition score	CVD risk score
Age	**.23**	**.18**	**-.39**	**.15**
Mean RT	-	**.67**	**-.50**	-.02
IIV_RT_		-	**-.26**	-.01
Cognition score			-	-.03

Bivariate correlations (Spearman’s rho and Pearson’s r) are shown for the full sample, significant findings shown in bold (*p* < .05).

In unadjusted Cox models, greater IIV_RT_ and slower mean RT were both individually associated with all-cause mortality (Table 3. Model 1: IIV_RT_ left side; Mean RT right side). The effect sizes were comparable for the two measures; the hazard was raised by approximately 35% for a 1 *SD* increase in variability or mean RT. However, in separate multivariable Cox regression models adjusting for global cognition and the other covariates (i.e., age, sex, APOE ɛ4 status, cardiovascular risk score), IIV_RT_ remained significant but mean RT did not reach significance (Table 3. Model 2: IIV_RT_ left side; Mean RT right side). When both measures were entered together in a basic regression model adjusted for age and sex, the effects were considerably attenuated (mean RT: Wald = 1.58 HR = 1.12 (95%CI: .94–1.31) *p* = .21; IIV_RT_: Wald = 1.42 HR = 1.15 (95%CI: .91–1.44) *p* = .23), indicating that the respective variables did not contribute uniquely above the other. A similar result was obtained when the remaining covariates were included in the model. This was not unexpected given the strong correlation of .67 between the two measures (see Table 2). To determine the best set of independent predictors, backward elimination was used from the fully adjusted model, with IIV_RT_, mean RT and all covariates entered at the first step (Table 3: Model 3). In this final model, IIV_RT,_ age, sex and cognition were retained as the most parsimonious set of predictors of all-cause mortality, noting that cognition was not a significant independent predictor in the model.

**Table 3:** Cox proportional hazards regression models of all-cause mortality over 8 years for IIV_RT_ (left side) and Mean RT (right side).

	Wald	HR	95% CI	*p*	Wald	HR	95% CI	*p*
***Model 1***								
IIV_RT_	13.0	1.35	(1.15–1.59)	**< .001**	-	-	-	-
Mean RT	-	-	-		22.63	1.36	(1.20–1.54)	**< .001**
***Model 2***								
IIV_RT_	4.09	1.22	(1.01–1.48)	**.04**	-	-	-	-
Mean RT	-	-	-	**-**	1.88	1.11	(.96–1.30)	.17
Age	45.92	1.13	(1.09–1.17)	**< .001**	46.20	1.13	(1.10–1.17)	**< .001**
Sex (male)	8.99	1.66	(1.19–2.31)	**.003**	8.24	1.62	(1.17–2.25)	**.002**
Cognition score	3.92	.87	(.76–1.0)	.05	2.80	.88	(.76–1.02)	.09
E4 (≥1 ɛ4 allele)	.13	.94	(.65–1.35)	.72	.06	.95	(.66–1.38)	.80
CVD risk score	1.36	1.03	(.98–1.09)	.24	1.44	1.03	(.98–1.09)	.23
***Model 3***								
Age	50.58	1.13	(1.10–1.17)	**< .001**				
Sex (male)	13.97	1.78	(1.32–2.42)	**< .001**				
Cognition score	3.50	.88	(.77–1.01)	.06				
IIV_RT_	4.00	1.22	(1.00–1.47)	**.045**				

Cox proportional hazards analyses were used to test each model. IIV_RT_ and Mean RT were examined separately for Models 1 and 2. The results presented on the left side of the table are for models examining IIV_RT_ and on the right side of the table for Mean RT. Model 1 measured predictive value of each RT measure unadjusted for covariates (N = 861; 191 deceased). Model 2 examined individual RT measure and all covariates together using enter method (N = 789; 172 deceased). Model 3 included IIV_RT_, mean RT and all covariates using the backward step (Wald) procedure (N = 789; 172 deceased). Final model is shown.

RT = reaction time; IIV_RT_ = intra-individual variability of reaction time.

Sex represents the risk of mortality for males relative to females. Age is measured in years. Cognition score is a global composite score obtained from average performance on 10 neuropsychological measures. CVD risk score is based on a Framingham-type composite score.

Mean RT, IIV_RT_ and global cognition measures were analyzed per standard deviation unit. p-values in bold indicate significance at 0.05 level.

Next, in order to account for the potential effects of dementia pathology on the associations of RT measures and mortality, we evaluated the determinants of survival in persons free of dementia. Over the 8-year study period, 82 individuals from our initial cohort of dementia-free elders, received a consensus diagnosis of dementia from the expert panel. Three further participants with evidence of cognitive impairment while in the study and dementia listed as the cause of death on death certificates were also classified as incident dementia cases. Altogether 29 of 191 of decedents (15.2%) had a dementia diagnosis. Separate multivariable Cox regression analyses were repeated after removing these 85 participants with incident dementia from the baseline cohort (*N* = 708; 144 deceased). The predictive strengths of the RT measures were virtually unchanged though neither attained conventional significance level after accounting for all risk factors (mean RT: Wald = 2.84 HR = 1.20 (.97–1.48) *p* = .09; IIV_RT_: Wald = 2.84 HR = 1.20 (.97–1.47) *p* = .09); age and sex were the only significant predictors in both RT models. Repeating the backward regression after eliminating dementia cases, with both RT measures and all covariates entered at the first step, IIV_RT_ remained a significant predictor in the final model along with age and sex (IIV_RT_: Wald = 3.89 HR = 1.23 (95%CI: 1.00–1.50) p = .049), global cognition was excluded. Additional data for full models with dementia cases excluded are provided in S1 Table.

## Discussion

In this large community-based old age cohort, greater variability in RT performance but not slower mean RT predicted all-cause mortality while adjusting for conventional mortality risk factors of age, sex, cardiovascular risk and APOE ɛ4 status and important potential confounders of low global cognition and prodromal dementia, both known to be associated with greater IIV_RT_ [4, 35] and increased mortality risk in old age [12, 13, 36]. Following removal of known incident dementia cases, the association between IIV_RT_ and time to death in the multivariable model decreased slightly and failed to reach significance. However, the findings remained robust in the most parsimonious model even after dementia cases were removed with greater IIV_RT,_ older age and male sex significant predictors of all-cause mortality.

Our findings broadly support and extend the small extant literature [9–11, 37] by providing further support for a strong association between IIV_RT_ and all-cause mortality having adjusted for a broad range of potential confounders. Previous studies have failed to adequately account for effects of overall cognitive level and dementia on the relationship between IIV_RT_ and mortality. Of the few studies that have included measures of cognition, investigators have simply controlled for performance accuracy on the individual tasks from which RT data were taken [9], or compared RT measures with one or two other cognitive measures (e.g., memory, visuospatial reasoning)[11]. The global cognition score employed in the present study is a more reliable and valid measure of cognition than those previously used since it is based on a large number of psychometrically validated cognitive tasks and it measures five major cognitive domains thereby more broadly capturing the individual’s level of cognitive function.

Dementia was also considered a potential confound in light of our previous work showing that incident dementia is associated with worse performance on cognitive measures, and IIV_RT_ and mean RT [21] and reduced survival time [38]. While no MAS participant had dementia at baseline, 85 were found to have dementia on biennial assessments over the 8-year follow-up period. After removal of these 85 incident dementia cases, the effect strength was slightly reduced (IIV_RT_: HR from 1.22 to 1.20) and failed to reach significance at conventional levels in the full model (*p* = .09), possibly because of the smaller sample size and loss of power, and higher variability scores in those who later developed dementia. However, when the model was refined by removing non-significant covariates, IIV_RT_ was retained in the set of best predictors, along with age and sex. Our findings suggest therefore that prodromal dementia does not explain the association of IIV_RT_ and time to death and address the shortcomings of previous studies that have either not considered dementia, even in the oldest-old when risk of dementia is very high ([e.g. 11], whose participants were aged up to 94), or have used less rigorous methods of controlling for dementia. For example, MacDonald and colleagues [9] relied solely on death certificates which listed “questionable dementia” as an antecedent condition (not primary cause) of death and reported “identical patterns of inference” with and without 33 cases of *questionable* dementia (12.5% of decedents). Batterham et al [10] used a cut-score of ≤24 on the MMSE to control for possible preclinical dementia. However, conclusions about the effects of dementia on the IIV_RT_-mortality relationship were limited by the low positive predictive value of the MMSE for dementia in population studies [39] and its crude measurement of global cognition compared to a neuropsychological battery and clinical consensus such as that used in the present study which is the gold standard for dementia diagnosis.

There is preliminary support for a stronger relationship of mortality with variability measures over mean RT measures when examined together [9, 10] although this is not universal across studies [37] or cognitive tasks [9, 10]. In our study, mortality effects were attenuated when both measures were included in the same base model and neither of the RT measures was an independendent predictor over the other. This may not be surprising given the strong correlation between the measures; increased IIV_RT_ may be reflecting a higher number of slow responses would also give rise to a slower mean RT. Furthermore, there is a part-whole association between IIV_RT_ and mean performance given that IIV_RT_ scores reflect residual variance after controlling for age-group and time on task (trial order) while mean RT includes all sources of variability [9]. However, it is noteworthy that IIV_RT_ was more weakly correlated with the global cognition score (r = -.26) relative to that of mean RT (r = -.50) supporting the premise that IIV_RT_ is capturing unique information relating to mortality that other cognitive measures are not tapping. From a cognitive perspective, increased IIV_RT_ is thought to reflect a) momentary fluctuations in attentional and executive control [40, 41]; b) individual differences in the rate at which task-related information accumulates to reach a critical threshold before triggering a response [42] or c) increased neural noise as the signal to noise ratio in the brain decreases[43]. The consequence in behavioural terms is increased variation in moment-to-moment processing efficiency uniquely captured by IIV_RT_ measures.

The present study suggests that variability from performance-based measures of reaction time is an independent risk factor and not simply a corollary of general cognitive decline or neuropathological disturbances associated with dementia. Therefore, other explanatory mechanisms for the relationship between within-person variability and mortality should be considered. At the neurobiological level, research suggesting the involvement of striatal dopamine D2 receptor binding in IIV_RT_ [8] is consistent with the theory that IIV_RT_ may reflect increased neural noise. Specifically, it has been postulated that neural noise increases with age as a result of reduced efficiency of the central nervous system and alterations in neurotransmitter systems (e.g., dopaminergic system) leading to more erratic processing which is captured by IIV_RT_ measures [43]. This neurobiological disturbance may become further exaggerated closer to death [10].

However, it is also possible that behavioural variability is a proxy measure of general age-related deterioration of physiological (body) processes. A deteriorating brain may simply be a part of a deteriorating body [37] as suggested by the “common cause” hypothesis of cognitive ageing [44] which delineates a single ageing process in physical and cognitive functions. Our study suggests that estimating variability from reaction time measures is a more sensitive indicator of survival than estimating mean levels of performance across several performance-based measures of reaction time and other cognitive domains although all are indicators of a degenerating brain. This does not necessarily argue against a common cause since different parts of the brain may react differentially, for example the hippocampus is more sensitive to hypoxia. Likewise, the frontal cortex is more vulnerable to aging processes [45] and this may underlie the sensitivity of IIV_RT_ measures to impending mortality. Future research using a multimodal approach incorporating cognitive and physical measures, and imaging to examine trajectories of intra-individual variability, physical frailty and neurodegeneration, and their associations with mortality in old age, may shed light on the common cause hypothesis.

Limitations of the research should be considered. The primary focus of our study was on cognition and inclusion of a comprehensive set of potential predictors of mortality is beyond the scope of this study. The selection of variables for the current study was based on the most commonly examined in the literature. In a recent publication, our group has examined a comprehensive range of risk factors for mortality, and also for dementia and Mild Cognitive Impairment [46]. We identified 85 cases with dementia over the follow-up period corresponding to an incidence rate of 19.1/1000 person-years in this older adult community sample. Although this is in line with published rates globally [47, 48], some cases of dementia may not have been detected before death. There are several reasons for this. First, the study design used 2-year assessment intervals and conversion to dementia may have occurred after the last assessment but before death. Second, a common issue in longitudinal studies is the problem of attrition and persons who drop out of observational cohort studies have an increased likelihood of progression to dementia [49]. Third, in Australia we do not have access to a dementia registry, which may have allowed more complete identification of new dementia cases. Given these considerations, we may have underestimated the effects of dementia on the association of mortality and IIV_RT_. Despite these limitations, the present study represents the most rigorous attempt to date to control for general cognitive level and dementia, and also major risk factors using comprehensive assessments and diagnoses based on clinical criteria. Nonetheless, it is important that future research address the possibility of dementia and other risk factors affecting the association of IIV_RT_ and mortality.

In conclusion, the present study suggests that variability of RT represents a unique cognitive marker of impending death beyond an individual’s overall cognitive function and various other risk factors. Accounting for new cases of dementia up to 8 years after the baseline assessment did not alter the IIV_RT_-mortality association suggesting that this association is independent of dementia-related neuropathological changes in the prodromal period. The findings supports the view of IIV_RT_ as a behavioural marker of neurobiological integrity [22] and that this may underlie its sensitivity to terminal decline and death, as well as other neuropathological states such as dementia [21], and falls risk [50].

## Supporting information

S1 Table Cox proportional hazards regression models of all-cause mortality over 8 years; 85 cases of incident dementia excluded. (DOCX)
