# Supplementary material for: Is intraindividual reaction time variability an independent cognitive predictor of mortality in old age? Findings from the Sydney Memory and Ageing Study
Source: PLoS One. 2017 Aug 9;12(8):e0181719. doi: 10.1371/journal.pone.0181719 (PMC5549897; doi:10.1371/journal.pone.0181719)
Supplement: S1 Table — (DOCX) [file pone.0181719.s001.docx]

Is intraindividual reaction time variability an independent cognitive predictor of mortality in old age? Findings from The Sydney Memory and Ageing Study

Nicole A. Kochan^1, 2*^, David Bunce^1,3^, Sarah Pont^1^, John D. Crawford^1^, Henry Brodaty^1,4^, Perminder S. Sachdev^1,2^

1. Centre for Healthy Brain Ageing (CHeBA), School of Psychiatry, University of New South Wales (UNSW) Australia, Sydney, NSW, Australia;
2. Neuropsychiatric Institute, Prince of Wales Hospital, Randwick, NSW, Australia;
3. School of Psychology, Faculty of Medicine and Health, University of Leeds, Leeds, UK
4. Dementia Collaborative Research Centre – Assessment and Better Care (DCRC-ABC), School of Psychiatry, UNSW Australia, Sydney, NSW, Australia;

*Corresponding author: [n.kochan@unsw.edu.au](mailto:n.kochan@unsw.edu.au) (NK)

S1Table:

Cox proportional hazards regression models of all-cause mortality over 8 years; 85 cases of incident dementia excluded.

|  | Wald | HR | 95% CI | *p* | Wald | HR | 95% CI | *p* |
| --- | --- | --- | --- | --- | --- | --- | --- | --- |
| *Model 1* |  |  |  |  |  |  |  |  |
| IIV_RT_ | 2.84 | 1.20 | (.97 - 1.47) | .09 |  |  |  |  |
| Mean RT |  |  |  |  | 2.83 | 1.20 | (.97-1.48) | .09 |
| Age | 45.88 | 1.15 | (1.10 - 1.19) | **<.001** | 50.84 | 1.15 | (1.11-1.19) | **<.001** |
| Sex (male) | 8.82 | 1.72 | (1.20 - 2.45) | **.003** | 8.98 | 1.73 | (1.21 - 2.48) | **.003** |
| Cognition score | 2.70 | .88 | (.76 - 1.03) | .10 | 1.39 | .91 | (.77 - 1.07) | .24 |
| E4 (≥1 ɛ4 allele) | .49 | .86 | (.56 - 1.31) | .48 | .06 | .52 | (.56 - 1.31) | .86 |
| CVD risk score | .86 | 1.03 | (.97 – 1.09) | .35 | .92 | 1.03 | (.97 - 1.09) | .38 |
| *Model 2* |  |  |  |  |  |  |  |  |
| Age | 71.17 | 1.16 | (1.12 – 1.20) | **<.001** |  |  |  |  |
| Sex (male) | 11.78 | 1.79 | (1.28 – 2.49) | **.001** |  |  |  |  |
| IIV_RT_ | 3.89 | 1.23 | (1.00 – 1.50) | .049 |  |  |  |  |

Cox proportional hazards analyses were used to test each model. Model 1 measured predictive value of each RT measure adjusted for covariates in separate analyses using the enter method (*N*=708; 144 deceased). Model 2 used the backward step (Wald) procedure and included IIV_RT_ and mean RT, and all covariates (*N*=708; 144 deceased). Final model is shown.

RT = reaction time; IIV_RT_=intra-individual variability of reaction time.

Sex represents the risk of mortality for males relative to females. Age is measured in years. Cognition score is a global composite score obtained from average performance on 10 neuropsychological measures. CVD risk score is based on a Framingham-type composite score.

Mean RT, IIV_RT_ and global cognition measures were analyzed per standard deviation unit. *p*-values in bold indicate significance at 0.05 level.
